# Single-Cell Analysis in the *Omics* Era: Technologies and Applications in Cancer

**DOI:** 10.3390/genes14071330

**Published:** 2023-06-24

**Authors:** Michele Massimino, Federica Martorana, Stefania Stella, Silvia Rita Vitale, Cristina Tomarchio, Livia Manzella, Paolo Vigneri

**Affiliations:** 1 Department of Clinical and Experimental Medicine, University of Catania, 95123 Catania, Italystefania.stel@gmail.com (S.S.);; 2 Center of Experimental Oncology and Hematology, A.O.U. Policlinico “G. Rodolico—S. Marco”, 95123 Catania, Italy; 3 Humanitas Istituto Clinico Catanese, University Oncology Department, 95045 Catania, Italy

**Keywords:** single-cell analysis, CTCs, omics, bioinformatic approaches, cancer, precision medicine

## Abstract

Cancer molecular profiling obtained with conventional bulk sequencing describes average alterations obtained from the entire cellular population analyzed. In the era of precision medicine, this approach is unable to track tumor heterogeneity and cannot be exploited to unravel the biological processes behind clonal evolution. In the last few years, functional single-cell *omics* has improved our understanding of cancer heterogeneity. This approach requires isolation and identification of single cells starting from an entire population. A cell suspension obtained by tumor tissue dissociation or hematological material can be manipulated using different techniques to separate individual cells, employed for single-cell downstream analysis. Single-cell data can then be used to analyze cell–cell diversity, thus mapping evolving cancer biological processes. Despite its unquestionable advantages, single-cell analysis produces massive amounts of data with several potential biases, stemming from cell manipulation and pre-amplification steps. To overcome these limitations, several bioinformatic approaches have been developed and explored. In this work, we provide an overview of this entire process while discussing the most recent advances in the field of functional *omics* at single-cell resolution.

## 1. Introduction

Over the last few decades, major steps forward have been made in the understanding of cancer biology, including disease pathogenesis, progression and dissemination to distant sites. This deeper insight has allowed several advances in cancer diagnosis and treatment, leading to a paradigm shift from a “one size fits all” to a “tailored” approach [1]. Despite this relevant progress, metastatic cancer patients still cannot be cured. The clonal heterogeneity of tumor cells, developing during the natural course of the disease and especially under pharmacological selective pressure, represents the main hurdle for cancer eradication. Indeed, spatial, and temporal heterogeneity acquired by tumor subclones confer on them evolutional advantages and resistance to multiple therapies [2,3].

Standard cell-based assays measure the average response in a bulk population, assuming that this average value is representative of each neoplastic cell, or at least the majority of cancer cells. This assumption excludes the possibility that a small but crucial cell subpopulation can be lost. The study of individual cell heterogeneity is critical to detect these subpopulations, and analysis at the single-cell level can disclose molecular mechanisms which cannot be detected in the bulk population. Single-cell analysis also makes it possible to generate a molecular fingerprint representing the specific functional state of any given cell [4]. Moreover, tracing tumor heterogeneity is challenging and requires longitudinal follow-up over time. In this context, liquid biopsy, i.e., the identification of “malignant products” (DNA, RNA, extracellular vesicles or entire cells) in the bloodstream of patients, is becoming a pivotal tool for intra-tumor heterogeneity investigation [5], especially for identification and study of circulating tumor cells (CTCs), at the single-cell level. CTCs provide an important opportunity to dissect cancer features and evolution not only at the genomic level, but also with regard to transcriptomics, proteomics, epigenomics and metabolomics [5,6]. Indeed, CTCs are rare tumor cells that, by the epithelial–mesenchymal transition (EMT), acquire the ability to detach from the primary tumor. This peculiar biological characteristic enables CTCs to enter the bloodstream, strongly increasing the risk of developing distant metastases [5,6]. Thus, investigation of molecular features at the single CTC level is critical to better understand the intra-tumor heterogeneity of each patient [7].

While at a *multi-omics* level, different technologies have been developed for single-cell isolation and *omics* analysis, there is also an increasing need for appropriate bioinformatic tools that will store, analyze and interpret the impressive amount of data deriving from a single cell [8,9,10,11]. Artificial intelligence and neural networks or deep learning approaches can be exploited for this purpose, and several findings are already available on this topic [12,13].

In this review, we will initially illustrate the consolidated cutting-edge technologies required for single-cell isolation and analysis and will then focus on the necessary bio-informatic tools needed for data storage and interpretation in the field of single-cell *omics*.

## 2. Single-Cell Isolation

**Figure 1:**
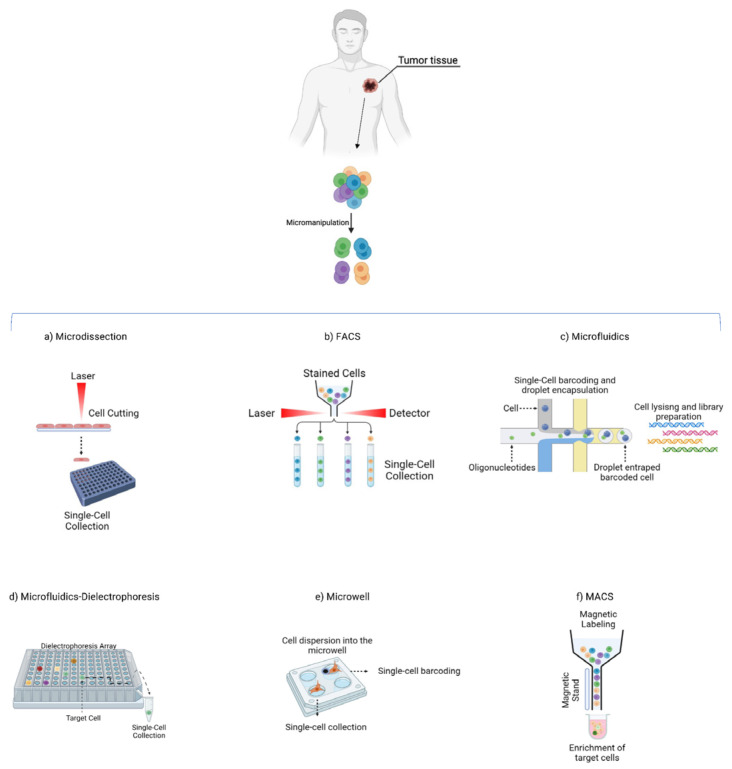
Single-cell isolation methods. (**a**) Microdissection: each cell is isolated by laser cutting and collected in a tube. (**b**) Fluorescence-activated cell sorting (FACS): a cell population is stained by specific fluorophore-associated antibodies and the desired single cells are separated and collected through a fluorescence-measuring detector. (**c**) Microfluidics: each cell is entrapped in a droplet and barcoded by one or more nucleotides. (**d**) Microfluidics–dielectrophoresis: cells are resuspended in a liquid and dispersed into a chip with a dielectrophoresis array. Cells entrapped in a cage are then moved by a non-uniform electric field pattern until each cell is collected in a tube. (**e**) Microwell: a cell population is dispersed into a chip containing microwells capable of entrapping individual cells. Each entrapped cell can be collected or barcoded. (**f**) Magnetic-activated cell sorting (MACS): cells are separated by magnetic beads through an antibody interaction with specific surface markers on the target cells. This technique allows sample enrichment but cannot perform single-cell isolation. Thus, the enriched sample needs to be further processed employing suitable methods for single-cell separation. In all approaches, each isolated single cell can be used directly for downstream analysis.

The identification of specific subtypes of selected cells followed by their individual isolation are mandatory steps in the context of single-cell analysis. In the following sections, we will discuss methods employed for single-cell isolation (Figure 1, Table 1).

### 2.1. Laser Microdissection

Laser microdissection (LMD)-based systems are high-resolution methods that allow the recovery of selected cells from their surrounding tissues using a microscope, without cell-labeling or genetic manipulation. These cells can then be used for a variety of downstream applications, including *omics* analyses [27].

To date, several systems are available, including laser capture microdissection (LCM), laser-assisted mechanical tissue microdissection (LAM), laser pressure catapult (LPC) microdissection, gravity-assisted microdissection (GAM) and mechanical microdissection (MMD). Employing these systems, the cells of interest are manually selected on a screen and a laser is then used to precisely cut tissue for subsequent cell isolation [10].

The LCM system (Figure 1a) is the most frequently used method [14,15]. The main advantage of LCM is its precision and versatility, which allows the obtaining of pure target cells from a wide range of tissues while preserving the morphological features of both the chosen cells and the residual tissue. Hence, it is possible to work on the remaining specimen after removing the selected cells. Furthermore, LCM can be exploited for both fresh and fixed tissues [4,28]. Still, LCM may generate technical artifacts, such as DNA or RNA damage caused by the ultraviolet laser beam [29].

### 2.2. Fluorescence-Activated Cell Sorter (FACS)

Fluorescence-activated cell sorter (FACS) is a high-throughput and sensitive technique that allows fast sorting and quantitative detection of cells, achieving a purity higher than 98% [16]. Each cell can be simultaneously labeled with multiple antigens and isolated according to granularity, size and fluorescence (Figure 1b). Several studies demonstrated that rare-cell populations, including CTCs, can be detected in liquid biopsies using FACS [9,17,18]. Given its high specificity and sensitivity, this approach can be used for single-cell isolation when the marker of interest is expressed at low levels [4,19,20]. However, drawbacks of this technique are the need for a high starting number of suspended cells (i.e., input >10,000 cells), the potential damage of fixative solutions required for intracellular antibody staining and the reduced viability of sorted cells due to the rapid flow in the machine [9].

### 2.3. Microfluidics

Microfluidics is a technology that sorts cells by employing several approaches, all based on physical and chemical cellular characteristics [9].

Recently, droplet microfluidic methodology has emerged as an extremely promising technology. This approach combines the flow rates of one cell with a cell partitioning system by chemical particles (barcoding), resulting in a barcoded single-cell capture (Figure 1c). Barcodes will then be used for library generation [21,22]. An advanced droplet-based single-cell method is the split-pool barcoding. It achieves single-cell resolution by combining several barcodes (combinatorial indexing). Using this approach, it is possible to profile millions of cells in one single experiment [23]. An advanced microfluidic-based method is the dielectrophoresis array-based system. This technology combines microelectronics and microfluidics in an automated platform to isolate pure single cells [24]. This technology relies on dielectrophoresis, i.e., the ability of a nonuniform electric field to exert forces on individual cells. Thanks to these electric forces, each cell is individually trapped in separate cages and independently captured by the device (Figure 1d).

### 2.4. Microwell-Based Systems

Using microwell-based single-cell isolation methodologies, the bulk cell population is dispersed into a microarray containing micropores capable of trapping one cell (Figure 1e). Once the single cell is entrapped in the well, it can be barcoded and then used for library preparation. Alternatively, single cells may be collected and later used for specific downstream analyses [23].

### 2.5. Magnetic-Activated Cell Sorting (MACS)

Magnetic-activated cell sorting (MACS) uses a passive separation technique, ensuring high-throughput separation of magnetically labeled cells through antibody-coated magnetic beads [9].

**Figure 2:**
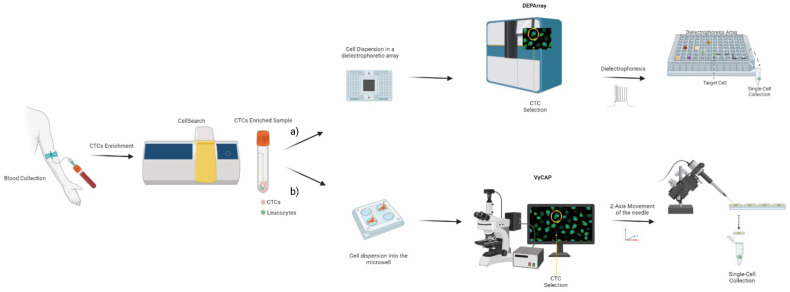
Liquid biopsy workflow proposed for single CTC isolation. Blood is collected and processed by the CellSearch system to obtain an enriched sample used to isolate single CTCs. Two approaches can be used. (**a**) The CTC bulk population is dispersed in a liquid onto a chip with a dielectrophoresis array. Cells are then selected by immunofluorescence images and isolated employing a microfluidic–dielectrophoresis system (DEPArray). (**b**) CTCs are dispersed in a chip containing microwells that entrap one cell per well. Each cell image is acquired by an immunofluorescence microscope, selected and isolated by a puncher system (VyCAP puncher system).

The cells are separated by magnetic beads through an antibody interaction with specific surface markers on the target cells (Figure 1f). Two types of MACS separation methods are possible via positive or negative selection of target cells. In the positive selection, the target cells are recognized by the antibody-coated beads and the remaining non-targeted cells are washed out [25]. In the negative selection, the unwanted cells are recognized by magnetic beads and the remaining target cells are transferred and collected. The disadvantages of this technique are that (i) cells could be damaged due to their interaction with the magnet stand, and (ii) MACS can only separate the entire cell population. Thus, a second approach is necessary for single-cell isolation [4]. The MACS system is exploited by the Food and Drug Administration (FDA)-approved CellSearch instrument [26], which is used to enumerate CTCs from metastatic breast, prostate and colorectal cancer patients with prognostic implications [30,31,32]. The immunomagnetic selection is performed by epithelial cell adhesion molecule (EpCAM) ferrofluids conjugated antibodies, followed by CD45, cytokeratin and DAPI staining. The CTCs-enriched sample can be processed to isolate single CTCs using specific instruments, including the DEPArray or VyCAP. DEPArray is an instrument that collects single CTCs according to microfluidics and dielectrophoretic-based methods (Figure 2a) [33]. VyCAP is a microscope microwell-based single-cell isolation technology that automatically selects and isolates CTCs [34,35]. It is composed of a disposable device that distributes single cells into an “isolation chip”, where each cell is entrapped in a microwell. Subsequently, a puncher system automatically isolates single cells according to the z-axis movement of a specific needle that locates the cell in a tube (Figure 2b).

## 3. Methods for Functional Genomics at Single-Cell Resolution

**Figure 3:**
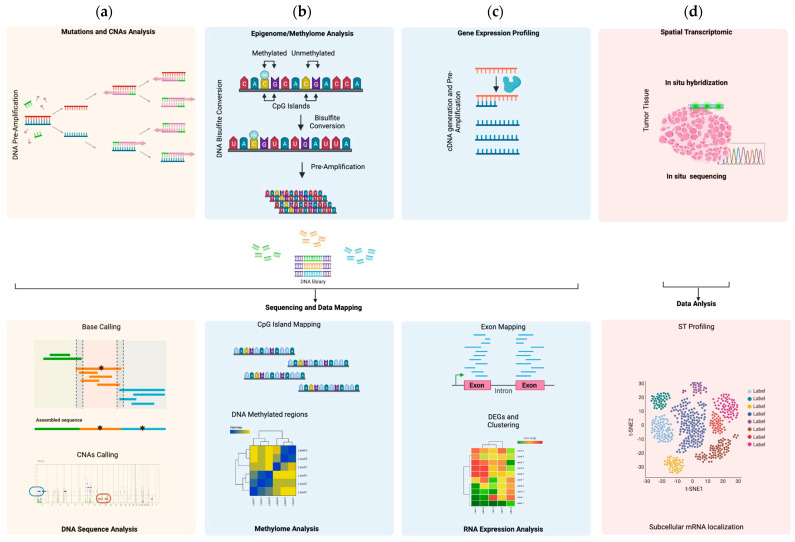
The principle of single-cell sequencing and analysis. (**a**–**c**). Collected single cells are lysed and analyzed for (**a**) mutations and CNAs: DNA is amplified, and the product is used for library preparation and sequencing. Using a specific DNA reference, the reads are aligned, and the mutations and CNAs are identified; (**b**) epigenome/methylome analysis: DNA is subjected to bisulfite treatment to convert unmethylated cytosines in uracil. In a pre-amplification step, uracil residues are converted in thymine. Libraries are then prepared and sequenced. The obtained reads are mapped using a specific DNA refence, and methylation levels are identified; (**c**) gene expression profiling: RNA is reverse transcribed in cDNA, pre-amplified and then used for library preparation and sequencing. The obtained reads will be mapped according to exome references, and DEG clustering will be performed; (**d**) picture showing the spatial transcriptomics principle. mRNA expression can be measured by both in situ hybridization and sequencing. The obtained data allow for the mapping of the expression profile of one cell in a specific coordinate.

In the last decade, several advanced techniques have been developed to analyze functional genomics, epigenomics and transcriptomics at single-cell resolution, promoting the single-cell *omics* era (Figure 3). Advancements in functional single-cell *omics* in cancer improved the understanding of intra-patient diversity, partially elucidating the biological mechanisms inducing tumor evolution, prognosis and drug resistance [36].

### 3.1. Single Cell Genomics

Single-cell genome analysis investigates and identifies genetic abnormalities, such as copy number alterations (CNAs) and nucleotide variations (NVs) (Figure 3a). One cell contains approximately 6 pg of DNA and 2 copies for each gene. Hence, traditional polymerase chain reaction (PCR) can introduce several errors, causing misinterpretation of an identified genetic abnormality [37]. To avoid this phenomenon, several PCR-based methods have been proposed, including linker-adapter PCR, interspersed repetitive sequence PCR (IRSPCR), primer extension pre-amplification PCR (PEP-PCR), multiple displacement amplification (MDA), looping-based amplification cycles identified as MALBAC and a MiCA-based centrifugal droplet generation technique with emulsion multiple displacement amplification [38,39,40,41,42,43].

### 3.2. Single-Cell Epigenomics

Single-cell epigenomics is a powerful new method to better understand gene expression regulation and cell–cell diversity in the context of tumor heterogeneity. Epigenetic events involve chemical modifications of both DNA and histone proteins, modulating chromatin access and transcription factors activity. DNA modifications mostly include the chemical addition of a methyl group on cytosine residues (metC), which can be mapped by sequencing of the bisulfite-converted DNA, generating genome methylation level data [44,45] (Figure 3b). For histone marker modifications, mapping is carried out by chromatin immunoprecipitation followed by sequencing [46].

### 3.3. Single-Cell Transcriptomics

Single-cell transcriptomics is an advanced technology that is currently employed to investigate the gene expression profile of an individual cancer cell. Unlike DNA, RNA presents multiple copies for each expressed gene, representing an advantage for gene profiling analysis. However, RNA is an unstable molecule, and its manipulation requires larger amounts of starting material. At the single-cell level, the amount of RNA is very low, therefore, after cDNA generation, a cDNA pre-amplification step is mandatory to perform single-cell transcriptomics (Figure 3c) [20,47,48]. The most reliable techniques include ScNaUniseq, MATQ-seq, Smart-seq2, CEL-seq2, MARS-seq, Fludigm C1 and 10× Genomics (Table 2) [22,49,50,51,52,53,54,55].

Among them, MARS-seq, SMART-seq2 and 10× Genomics are often used. MARS-seq represents an automatic method where single cells are sorted by FACS, then rapidly lysed, and a 3′ end-counting mRNA sequencing is applied for partial cDNA generation. Indeed, this method does not generate a full-length cDNA. At this time, cDNA is barcoded by a unique molecular identifier (UMI), which ensures the correct quantification of gene expression [53,54]. Smart-seq2 is a method which generates full length cDNA and gives higher coverage. The most important limitation is that UMIs cannot be incorporated in the cDNA strand [51]. Finally, the most popular single-cell RNA sequencing technique is 10× Genomics Chromium, developed by Zheng et al. [22]. The method entraps single cells in one droplet using a specific emulsion approach, and then each gel bead, containing barcoded oligonucleotides, UMIs and oligo-dTs, will trigger the conversion of poly-A-RNA in cDNA that will be used for library preparation.

More recently, single-cell spatial transcriptomics (scST) is emerging as an interesting tool for gene expression profiling analysis, including FFPE tissues. Spatial transcriptomics can measure gene expression according to coordinates of the cells within a tissue, representing a promising approach for biological investigation of cells’ transcriptomes in their native context [56]. This method is classified within imaging-based techniques, such as in situ sequencing or hybridization approaches (Figure 3d) [57,58,59,60]. Among them, spatial transcriptomics, released as Visium by the 10× Genomics platform, is widely used. Visium Spatial Gene Expression can establish if gene activity is occurring in a tissue and can be applied on both the whole transcriptome and specific sub-sets of transcripts. Hence, by whole transcriptome analysis, the method can identify a specific spatial organization for each cell type, while focusing on a specific pre-designed gene set is possible to analyze a given intracellular signaling associated with a targeted disease, including cancer [61,62,63]. An additional method concerns GeoMx, which uses spatial high-plex technology developed for the Nanostring platform [64,65]. Using this method, it is possible to quantify the protein or RNA indexing nucleotide assigned for each target. In addition, this technique is a non-destructive method; hence, each slide can be used for other analyses. Finally, an ongoing technique based on single-cell spatial transcriptomics is the multiplexed error-robust fluorescence in situ hybridization (MERFISH). MERFISH is an image-based spatial technology that can work at single-cell resolution by directly mapping a single cell in a given spatial position. Although MERFISH can only study hundreds to a few thousand genes, its ability to work at a single-molecule level and at subcellular spatial resolution makes it a method suitable for studying a target list of genes in a given spatial context [66,67]. However, MERFISH is a relatively new technique and comparative studies with other well-established methods are requested, especially for analysis at single-cell resolution.

Recently, a new methodology called RNA velocity has been proposed to predict the future transcriptional state of individual cells based on the time derivative of their gene expression [68]. RNA velocity creates the possibility of studying cellular transcriptomic processes, measuring spliced and un-spliced mRNA expression in a given cell. RNA velocity distinguishes pre-mRNA (un-spliced) from mature mRNA (spliced), estimating the change in the abundance of the two forms of RNA. When a positive velocity is detected, the un-spliced pre-mRNA isoform is increasing, an event that is followed by up-regulation of spliced mRNA. On the contrary, a negative velocity indicates the opposite process, with a consequent down-regulation of the spliced mRNA. The combination of velocities across genes is used to extrapolate the future state of an individual cell [67]. An interesting method, in the context of RNA velocity at a single-cell level, is represented by SIRV (Spatially Inferred RNA Velocity), which integrates data derived from spatial transcriptomics and scRNA-seq. Through this integration, SIRV elaborates RNA velocity vectors for each cell, mapping these vectors according to spatial coordinates within the tissue [69].

## 4. Single-Cell Data Analysis and Bioinformatic Approaches

Data analysis represents the most challenging step in the interpretation of single-cell *omics*. The first issue in single-cell bioinformatic analysis concerns the normalization of the observed coverage due to the errors introduced by pre-amplification steps in both DNA and RNA sequencing [70].

Genome sequencing enables the analysis of the entire genome (whole-genome amplification, WGA) or only of the exon regions (whole-exome sequencing, WES). Data concerning SNVs and insertions/deletions (INDELs) are filtered to calculate the mutation rate, while CNAs are organized as a matrix derived from the read counts of each analyzed DNA region [71,72,73]. For example, a compound Poisson model followed by an empirical Bayes estimator was used by Daley et al. to predict genome coverage of an ongoing deep sequencing experiment [74]. Similarly, Deger et al. performed a WGA by LM-PCR and proposed a Burrows–Wheeler Alignment (BWA-MEM)-based bioinformatic pipeline for CNA estimation in CTCs to assess intra-patient tumor heterogeneity [75].

Transcriptomics experiments generate a large number of reads from each examined gene, making interpretation extremely complex. Usually, transcriptomics data are normalized as transcripts per million bases (RPMK), and several computational approaches have been proposed to detect variations in gene expression [76,77,78,79]. These data are analyzed, removing the unwanted batch effects; then an RNA expression matrix is generated, containing differently expressed genes using raw counts on a logarithmic scale [80,81]. However, in the context of transcriptome analysis at the single-cell level, RPMK is not used, and other bioinformatic approaches to application of statistical models for single-cell RNA-seq analysis are normally preferred. Among them, generalized linear models (GLMs), including scTransform [82] and GLM-PCA [83], have been described. In detail, scTransform uses the Person residuals from negative binomial regression as inputs for standard dimensional reduction techniques, unlike GLM-PCA, which uses principal component analysis for data with Poisson-distributed errors. An interesting approach to scRNAseq data analysis in the context of spatial transcriptomics is represented by a bioinformatic tool called cellular (Cyto) spatial positioning analysis via constrained expression alignment (CytoSPACE) [84]. One of the major questions in the context of single-cell spatial transcriptomics concerns gene coverage, which often is not adequate to obtain a representative transcriptome profile when a single-cell resolution analysis is performed [85]. CytoSPACE showed a guaranteed improvement in noise tolerance and also mapped individual cells from a scRNA-seq atlas in a location, in both bulk or single-cell spatial transcriptomics datasets, across diverse platforms and tissues.

For epigenomic events, methylome alterations are the most studied phenomenon in cancer. The analyses of promoter methylated regions [86,87,88,89] are processed by a matrix that scores the proportion of cells in which the identified nucleotide is either methylated or unmethylated [90]. Recently, Uzun and colleagues developed SINBAD (a pipeline for processing single-cell bisulfite sequencing samples and analysis of data), a tool for single-cell methylation analysis [91]. By generating a methylation matrix, this tool allows pre-processing, read mapping and methylation quantification.

Overall, these observations highlight the crucial role of computational pipelines in single-cell data interpretation. To improve single-cell data analysis integration, bioinformatic methods based on individual cell machine learning (ML) have been generated and are likely to further improve the correct interpretation of these findings.

### 4.1. Machine Learning for Functional Single-Cell Omics Data Analysis

**Figure 4:**
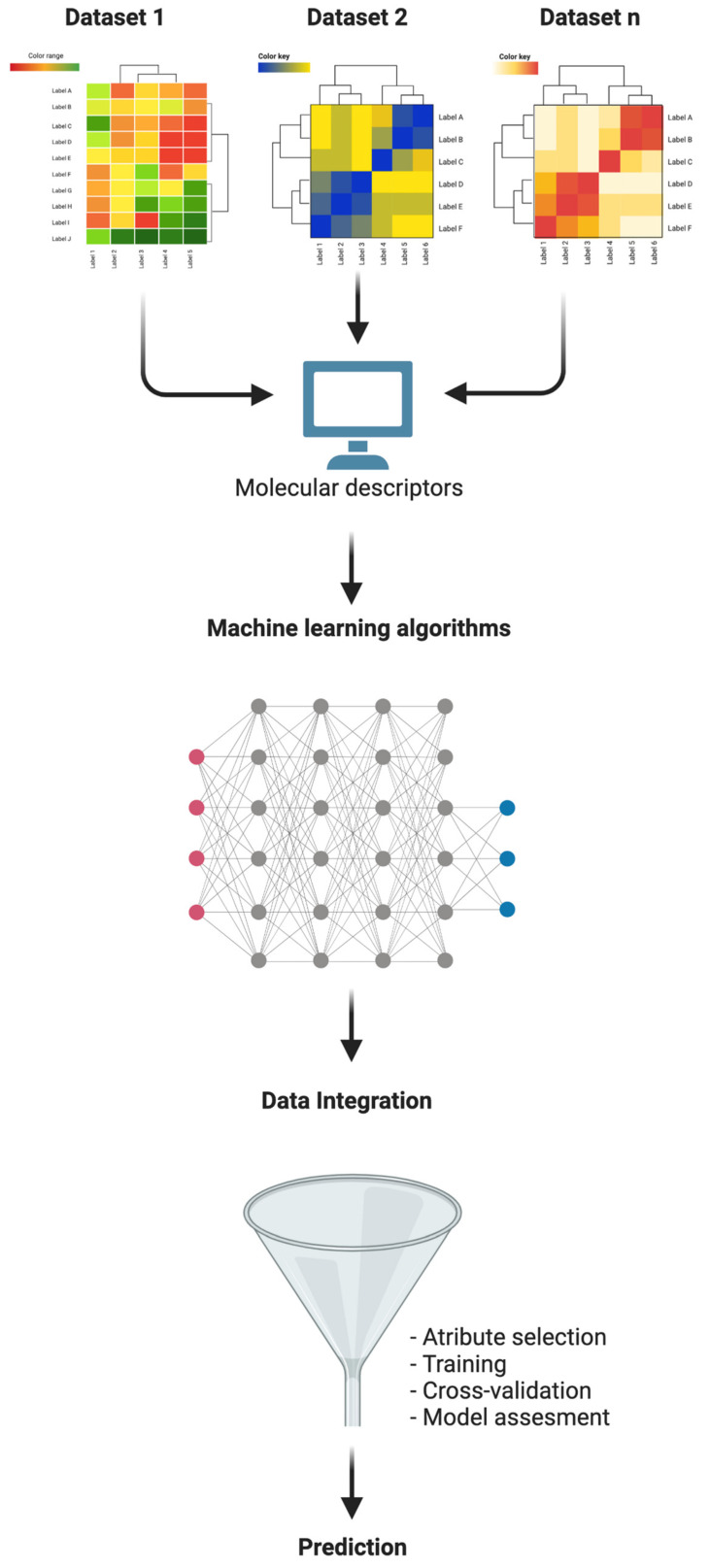
Machine learning approaches to single-cell omics data analysis. Different datasets are analyzed by bioinformatic methods. The obtained data are processed by machine learning algorithms, and data integration is performed to obtain a predictive model.

Machine learning is defined as a computational method that generates predictive models integrating and analyzing multiple datasets (Figure 4). In other words, ML is the study of models which can learn starting from obtained experimental data without a set of instructions [92]. In cancer, ML represents an innovative bioinformatic approach to investigate tumor evolution [93] and drug response [94] and to interpret sequencing data, including those deriving from single cells [13]. It can be exploited to integrate *multi-omics* data and identify pathways involved in cancer at the single-cell level [13,95,96,97]. Machine learning models comprise two groups, divided into unsupervised and supervised learning.

Unsupervised learning analyzes *multi-omics* data without mapping the input data to output data. The most used applications are (i) principal component analysis (PCA), which transforms high-dimension data into lower-dimension data while retaining as much information as possible [98]; (ii) canonical correlation analysis (CCA), which calculates how well different data matrices are correlated [99,100]; and (iii) multiple kernel learning (MKL), which transforms data into a higher-dimensional space, permitting an appropriate modeling [101].

Supervised learning is used to predict a specific event. Usually, given a specific input and output label, supervised learning can map the input data to the labeled information. In cancer, labeled information can be phenotype, disease-stage or cancer type. The most common supervised learning models are (i) elastic net, which is used to integrate *multi-omics* data with drug response [102]; and (ii) random forest [103], which is a non-linear ML model that elaborates complexity in the data and is useful in terms of prediction.

A bioinformatic workflow for scRNA-seq is normally performed by three steps concerning (i) the scRNA-seq data assembly, (ii) data processing and (iii) downstream analysis. Then, the obtained results can answer several biological questions. However, the interpolation of these complex data, in order to generate a predictive model, required an enhancement of artificial intelligence approaches. To this end, neural networks, an improvement to ML, have been developed as a root of deep learning (DL) algorithms, providing better predictive methods [12,104]. Deep learning mimics the brain’s neural functions in order to learn and generate a predictive model. Compared to standard ML, DL models provide a more powerful approach that better computes the heterogeneity associated with scRNA-seq data and technical noise [12,105,106]. This unquestionable peculiarity is linked to its neural network architecture. Basically, this model, inspired by human neurons, works by neuronal layers. Each layer receives information from previous layers, which, in turn, send information to the next layer. The input information, i.e., scRNA-seq data, that moves from one layer to each successor layer produces a learning model that can be trained for predictive model generation [107]. Several DL architectures have been proposed for single-cell *omics*. They include single-cell variation interference (scVI) and the single-cell autoencoder tool (scAEspy) [108,109]. Additional neuronal networks, such as Markov affinity-based graph imputation of cells (MAGIC) [110], variability-preserving imputation for accurate gene expression recovery (VIPER) [111], single-cell differential composition analysis (scDOC) [112] and neural network-imputation for single-cell RNA sequencing and cell-type clustering (NISC) [113], impute the drop-out probability of an RNA profiling experiment. Neural networks display better performance when compared to traditional ML, and several deep learning algorithms are progressively emerging [105,114,115,116].

In conclusion, compared to classical ML, DL has demonstrated a high capacity for computing massive amounts of data derived from scRNA-seq, and also for removing technical noise and artifacts derived from single-cell manipulation procedures. Although the results derived from this bioinformatic triumphing process can be used to answer relevant biological questions linked to transcriptomics, additional improvements are needed as a result of the high complexity of the data. Therefore, potential future directions in the field are oriented toward developing DL algorithms better able to compute the scRNA-seq-derived data to determine the crucial steps of transcriptome analysis and to create a more advanced pipeline.

### 4.2. Data Portals Comprising Multi-Omics Data

Published *omics* data are organized and classified by online portals (Table 3), including [i] the Cancer Genome Atlas (TCGA) [117], which collects data from 20,000 tumors; [ii] the International Cancer Genome Consortium (ICGC) [118], which incorporates projects derived from the TCGA; and [iii] the Catalog of Somatic Mutations in Cancer (COSMIC) [119,120] by the Welcome Sanger Institute, which collects multi-omics data from both cancer cell lines and primary tumors. Important single-omics databases include several consortiums, such as the single-cell eQTLgen [121], the Functional Annotation of the Mammalian Genome (FANTOM) [122] and Genotype-Tissue Expression (GTEx) [123]. eQTLgen was created to identify the genetic variants in specific immune cell types. This consortium also integrates genetic signatures on specific maps of human cells generated by the Human Cell Atlas (HCA), including collections, at the single-cell level, curated by the Broad Institute, and the Human Tumor Atlas Network (HTAN) funded by the National Cancer Institute [124]. HTAN is a three-dimensional atlas of cellular characteristics, such as morphological and molecular features of human cancer [125]. FANTOM and GTEx collect transcriptomics profiles of several human cells, including those at the single-cell level. Finally, OmniBrowser, an interactive analysis and visualization platform for large-scale multi-omics single-cell data, represents the world’s largest and most comprehensive single-cell transcriptome database URL: https://omnibrowser.abiosciences.com/#/home accessed on 21 May 2023.

Recently, scMethBank, a catalog of single-cell whole-genome methylation maps, has also been assembled by the Chinese National Center for Bioinformation. This database allows for the exploration of whole-genome methylation at the single-cell level [126].

Another important repository for multi-omics single-cell data is represented by the CellxGene platform (https://cellxgene.cziscience.com accessed on 21 May 2023) [127] and single-cell portal of the Broad Institute (https://singlecell.broadinstitute.org/single_cell accessed on 21 May 2023). CellxGene is a collection of tools useful for downloading and analyzing single-cell data. The Broad Institute portal contains around 500 studies, with data derived from more than 35 million cells. Using both of these online portals, scientists can explore a large single-cell dataset, including scRNA-seq, WGA and WGS associated with specific diseases and cell types. Moreover, it is possible to interpolate a specific gene with a given disease to identify a potential relationship.

Overall, multi-omics portals represent a suite of web-based platform tools that assists researchers in understanding and gaining a more comprehensive picture of the field of single-cell omics data.

## 5. Limitations and Pitfalls of Single-Cell Analysis

Here, we exposed single-cell analysis as an advanced approach to the study of several biological questions concerning single-cell resolution, as it is able to dissect intracellular heterogeneity, which is masked when a bulk analysis is performed. The most popular methods used to study the biology of one cell consist of analyzing the entire genome and transcriptome via *omics* approaches. The WGS method is the gold standard in the assessment of chromosome imbalances and CNVs, while whole-transcriptome analysis is a powerful approach to investigate cell-to-cell diversity in the context of adaptative stimuli such as microenvironment, drug exposure or tumor dissemination [128].

Despite these indisputable rewards, several limitations occur, caused by single-cell isolation procedures, the low amount of genetic material, the massive amount of data and technical-derived noise. Concerning single-cell isolation procedures, various methods have been discussed in the present work, and each of them aimed to isolate one cell from a tissue or circulating system. In all cases, the cell manipulation associated with each procedure might influence the cell viability and transcriptomic profiling, affecting both the number of viable cells and *omics* data [129]. Furthermore, although the single-cell analysis permits the identification of cell-to-cell diversity, when one cell is removed from tissue, it loses “*its spatial context*”. To address this issue, spatial transcriptomics was developed, which aimed to analyze the transcription state in a specific location of one given cell [130]. However, several advancements are needed, including the improvement of gene coverage, although in this regard, a new bioinformatic approach is emerging [84]. On the other hand, the low abundance of genetic material, DNA and RNA, constrains the pre-amplification steps, which, in turn, introduce crucial biases for the maintenance of both chromosome biallelic architectures and the gene expression ratio, especially for lower expressed genes [131,132]. Lastly, sequencing methods produce massive amounts of data, and a plethora of bioinformatic approaches have been developed to solve this question. However, several limitations, such as the distortion of data often associated with a limited number of cells not representative of the entire population, and a restricted number of potential combinations between them, do not allow the obtaining of precise information with respect to the biology of one cell [133].

An important limitation concerning the scRNA-seq at single-cell resolution is represented by the cell cycle state of a given cell in a given cell cycle phase. It has been well established that in moving between cell cycle phases, the transcriptomics arrangement is affected, thus impacting gene expression profiling. This cellular event leads to additional noise in subsequent data analysis and should be removed. A previous published study, Cyclum, used a DL approach to data obtained by Hoechst-stained cells to distinguish the cell cycle phases [134].

Overall, single-cell analysis remains an intriguing and fascinating approach to study tumor evolution, but a series of limitations make it subject to further improvement.

## 6. Application of Functional Single-Cell Genomics in Cancer

**Figure 5:**
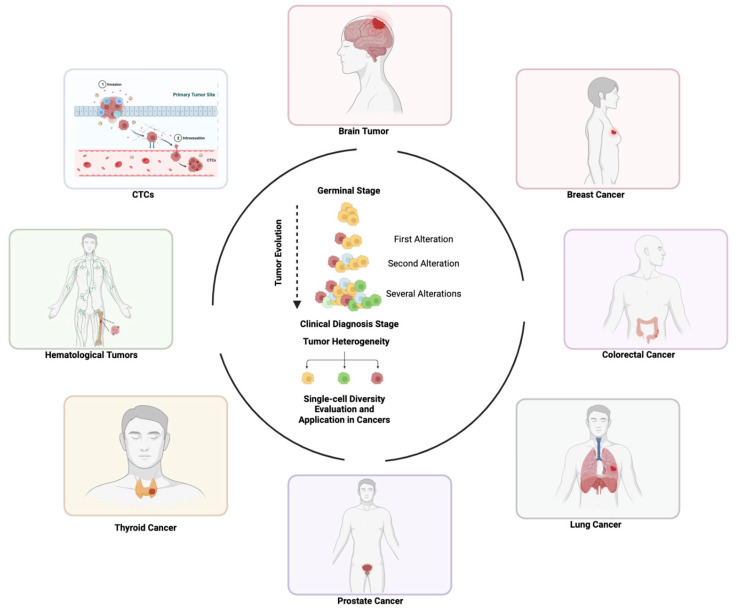
Single-cell analysis and application in cancer. Cancer develops from a germline stage. During its natural course, the disease acquires several alterations inducing tumor heterogeneity at diagnosis. Single-cell analysis, including CTCs, identifies cell-to-cell diversity, improving clinical management of cancer patients.

Single-cell sequencing has been widely applied in cancer research due to its ability to identify specific alterations in one cell, with relevant implications for cancer diagnosis and treatment (Figure 5). In this section we will provide an overview of available single-cell *omics* applications in different cancer types.

### 6.1. Brain Tumors

Primitive brain tumors classically derive from neoplastic transformation of glial cells, and their molecular profiling is mandatory for diagnosis [135]. Through single-cell mapping, several authors identified critical differences in the genomic and transcriptomic profiles of brain cancer cells, suggesting new potential therapeutic targets. Venteicher A.S. et al. dissected the intracellular heterogeneity at the single-cell level by comparing 14,226 scRNA-seq profiles with bulk RNA-seq derived from 165 patients, identifying a distinct tumor microenvironment composition that distinguished IDH-mutant oligodendrogliomas and astrocytomas [136]. The work published by Tirosh I. and colleagues documented different gene expression profile signatures of 4347 single cells on oligodendrogliomas, showing the classical IDH1/IDH2 dualism. The authors recognized two distinct profiles. The first one concerned a profile linked to transcriptome analysis, via the Smart-seq2 protocol [51], identifying signatures associated with cell proliferation which was enriched in rare subpopulations, traceable with cancer stem cells, known to support glioma cell growth. The second profile was derived from CNA analysis performed by the average expression of genes in large chromosomal regions within each cell. CNA analysis revealed distinct sub-clones associated with similar cellular hierarchies, assuming that the chromosome structure of oligodendrogliomas is strictly dependent on tumor clonal evolution [137]. Lasty, in the context of targeted therapy, single-cell data revealed *S100A4* as an immunoregulatory gene for suppressive T-cells, potentially representing an immunotherapeutic target in glioma patients [138].

### 6.2. Breast Cancer

Breast cancer can be classified in different intrinsic variants according to its molecular profiling [139,140,141,142]. For this reason, single-cell analysis is a powerful tool to study cell diversity and improve disease prognosis and treatment [143,144]. Using RNA sequencing, performed at the single-cell level by 10× Genomics, and unsupervised cell clustering employing PCA, Hu et al. demonstrated that basal-like estrogen-receptor-negative breast cancer might originate from luminal progenitors, while estrogen-receptor-positive disease likely stems from mature luminal cells [145]. Moreover, a polyclonal origin for ductal in situ and invasive breast carcinoma has been reported by Casasent and colleagues using single-cell DNA sequencing [146]. Interestingly, the authors developed topographical single-cell sequencing (TSCS) to address the limitation of single-cell isolation, which causes the loss of spatial information for each cell. TSCS combines LCM with WGA at single-cell resolution, preserving the spatial information, allowing the answering of critical questions about the invasive properties of ductal in situ carcinoma breast cancer. From these data, it emerged that genome evolution, truncal mutations and CNAs are initiating molecular events in an invasive process associated to co-migration of one or more tumor clones, that, while randomly escaping through the breakdown of the basement membrane, establish the invasive tumor mass. Lastly, although this study presents several limitations, including the small number of both patients and single cells analyzed, it highlights an interesting question on molecular mechanisms linked to the invasive properties of ductal in situ breast cancer.

### 6.3. Colorectal Cancer

Colorectal cancer (CRC) is classified in different subtypes according to its molecular profile, and each subtype shows differences in both clinical and biological properties that suggest high heterogeneity for this cancer type [147,148]. Considering the involvement of an immune response in this tumor, several authors mapped the T-cell immunoreceptor, applying single-cell technologies [149]. Zhang et al. reported a strong heterogeneity, identifying a state-transition relationship between T-cell populations and subpopulations differently distributed across the tumor tissue. Developing single T-cell analysis by RNA sequencing and TCR tracking (STARTRAC) indices (distribution, expansion, migration and transition), the authors, applying the unsupervised clustering analysis of scRNA-seq on 11,138 single T-cells, quantitatively described these indices as playing a crucial role in antitumor immunity via T-cells in CRC [150]. Methylation state is a crucial molecular feature in colorectal cancer [151,152]. Huang et al. profiled DNA methylation at the single-cell level, depicting a partial methylation domain (PMD) profile in more than half of the genome for each cell analyzed. This PMD covered mostly protein-coding regions, underlining a strong heterogeneity between the analyzed cells [153].

### 6.4. Lung Cancer

Lung cancer often displays a poor prognosis due to its rapid growth, invasive and early metastatic properties. A high heterogeneity is emerging in non-small-cell lung cancer (NSCLC) and small-cell lung cancer (SCLC) with the application of single-cell analysis. Using RNA-seq in individual NSCLC-derived single cells, Maynard and colleagues reported that cells surviving to treatment display a molecular signature associated with an alveolar cell pathway [154]. Furthermore, Wu reported high heterogeneity between NSCLC patients in both cellular composition and chromosome architecture [155]. Finally, in SCLC, the Yes-associated protein (YAP) and transcriptional coactivator PDZ-binding motif (TAZ) signaling were identified as crucial components of drug resistance and tumor heterogeneity [156].

### 6.5. Prostate Cancer

Prostate cancer is a common tumor in men worldwide. From a genomic standpoint, prostate cells have been classified as basal-like, luminal and neuroendocrine [157,158], highlighting cell-to-cell diversity in tumor tissue. At single-cell resolution, Song et al. showed high heterogeneity of tumor-associated epithelial cell states, including data obtained from organoids [159]. Moreover, clonal genomic and spatial diversity of prostate cancer cells was also documented by Chen and colleagues. They demonstrated that a subset of endothelial cells triggers cell-to-cell communication with tumor cells and that this phenomenon is enriched in castration-resistant prostate cancer, increasing cell invasion [160].

### 6.6. Thyroid Cancer

The most common thyroid cancer variant is the papillary subtype (PTC), which, although it often has an indolent course, may progress to an undifferentiated or anaplastic state, determining a dismal prognosis. Therefore, the identification of specific alterations that can predict disease progression or identify effective therapeutic targets can play a crucial role in the clinical management of these patients [161,162]. Pu et al. reported that single-cell transcriptomics analysis allowed for the identification of molecular markers indicative of disease progression that may translate into novel therapeutic approaches for these patients [163]. Finally, a transcriptome investigation by Wang and colleagues, limited to thyroid cancer patients displaying malignant tumors in both lobes, identified a high heterogeneity when comparing carcinomas in different lobes of the same thyroid gland [164].

### 6.7. Hematological Malignancies

Hematological tumors comprise a group of malignancies characterized by the clonal expansion of cells derived from the hematopoietic system [165]. Several authors reported the potential usefulness of single-cell analysis in hematological tumors. Ledergor and colleagues demonstrated that different gene expression profiles in single myeloma cells play a crucial role in proper patient diagnosis and treatment. The authors employed an automated massively parallel RNA single-cell sequencing framework (MARS-Seq), based on FACS sorting of single cells into well plates, and then subjected them to automated processing. The advantage of this approach is the high reproducibility, while the disadvantage is linked to the sorting method. Indeed, FACS required knowledge of specific surface markers; therefore, cells with unknown markers could not be isolated [166]. Interestingly, Hou et al. used a high-throughput single-cell sequencing approach based on WGE analysis employing MDA. The authors analyzed single-cell derived patients with *JAK2*-negative myeloproliferative disorders, documenting a monoclonal evolution of the tumor [167]. In chronic myeloid leukemia (CML), Zhang et al. used 10× Genomics single-cell RNA sequencing to predict imatinib resistance mechanisms. Through this approach, the authors raise an important question, implying that TKI resistance might be an intrinsic peculiarity of some CML patients associated with non-neoplastic immune cells, which can dissolve potential TKI efficacy in a given CML patient [168].

### 6.8. Circulating Tumor Cells

CTCs display both spatial and temporal heterogeneity when compared to the primary tumor. At the single-cell level, CTC *omics* analysis can be performed in several tumor subtypes [169], including prostate, breast, lung, thyroid and colorectal cancers [170,171,172,173,174,175]. Each isolated CTC can provide crucial information concerning the clinical and therapeutic scenario for any individual patient. Indeed, from each single CTC, it is possible to detect a spectrum of somatic mutations, affecting gene expression or copy number variations that may heavily inform further clinical approaches [7,176].

## 7. Summary and Future Outlook

In the era of personalized medicine, tumor molecular profiling is critical to define the peculiar features of each cancer type [177,178,179]. Cell-to-cell diversity plays a crucial role in the natural history of each malignancy, eventually influencing patient outcome. In the future, deciphering the cancer genome, transcriptome or epigenome using single-cell analysis can inform diagnostic and therapeutic decisions and refine patient management [180].

Despite the outstanding potential of this innovative approach, single-cell methodologies still pose many challenges. Indeed, they are complex techniques due to the low amount of starting material, ingle-cell manipulation, as well as gathering enough isolated cells to generate representative data. Nucleic acid amplification steps are mandatory, but they may introduce biases that must be considered during single-cell data interpretation. Hence, this technical noise can affect single-cell data analysis. To improve the overall performance of this approach, several bioinformatic pipelines have been proposed and implemented, including ML and more advanced neuronal networks or DL.

While *omics* data collected at the single-cell resolution likely represent the next frontier of molecular cancer research, further investigations are needed to improve these approaches and incorporate them into the clinical management of cancer patients.

## Figures and Tables

**Table 1:** Overview of systems and methods for single-cell isolation.

System	Method	Advantage	Limitations	References
Microdissection	Laser dissection of desiderate cell	High-resolution No previous manipulation of the tissue No damage for adjacent tissue	UV laser can damage the nucleic acids structure	[14,15]
Facs	Cells are stained and sorted by fluorescence detector	Sensitive including rare cells	Requires tissue dissociation can damage the cell Requires high number of target cells Requires specific cell staining	[9,16,17,18,19,20]
Microfluidics	Cell is captured by flow rate according to their physical and chemical features	High-resolution Use of barcodes to start directly with library preparation Can be image-based improving the selection of desiderate cell	Requires tissue dissociation can damage the cell Requires specific cell staining	[9,21,22,23,24]
Microwell-based System	Cells are dispersed into the microwell	High-resolution Use of barcodes to start directly with library preparation Can be image-based improving the selection of desiderate cell	Requires tissue dissociation can damage the cell Requires specific cell staining	[23]
Macs	Immunogenetic staining	Negative and positive selection	Requires tissue dissociation can damage the cell Only cell enrichment No single-cell isolation The cell suspension needs to be processed further to obtain single separated cell	[9,25,26]

**Table 2:** Overview optimal methodology for single-cell transcriptomics.

Data Portal	cDNA Generation	UMI	References
SCNA UMI-seq	Full length	YES	[50]
MATQ-seq	Full length	YES	[51]
10X GENOMICS	3′-end	YES	[22]
CEL-seq2	3′-end	YES	[53]
SMART-seq2	Full length	NO	[52]
MARS-seq	3′-end	YES	[54]
Fluidigim C1	3′-end	YES	[55,56]

**Table 3:** Overview of data portal for accessing to multi-omics data.

Data Portal	Source	Links	References
TCGA	Data from 20,000 tumors	URL: https://portal.gdc.cancer.gov/	[117]
ICGC	Incorporates data from TCGA	URL: https://dcc.icgc.org/	[118]
COSMIC	Primary tumors and cell lines	URL: https://cancer.sanger.ac.uk/cosmic	[119,120]
SINGLE-CELL eQTLGen	Genetic variations of immune cells and integrate data derived from HCA and HTAN	URL: https://eqtlgen.org/sc/	[121]
FANTOM GTEx	Transcriptomics profiling of human cells	URL: https://fantom.gsc.riken.jp/ https://www.gtexportal.org/home/	[122,123]
HTAN HCA	Genetic signature	URL: https://humantumoratlas.org/ https://www.humancellatlas.org/	[124,125]
scMethBank	A database of single-cell methylation maps for human and mouse	URL: https://ngdc.cncb.ac.cn/methbank/scm/	[126]
CellxGene	Collection of tools useful for downloading and analyzing of single-cells data	URL: https://cellxgene.cziscience.com	[127]
Broad Institute	A database of single-cell omics data	URL: https://singlecell.broadinstitute.org/single_cell	/

## Data Availability

Not applicable.
